# Supplementary material for: Polysaccharides of Salsola passerina: Extraction, Structural Characterization and Antioxidant Activity
Source: Int J Mol Sci. 2022 Oct 29;23(21):13175. doi: 10.3390/ijms232113175 (PMC9657462; doi:10.3390/ijms232113175)

Supplementary materials

Figure S1. ^{1}H spectra of the **SO1-1(a)**, **SO1-2(b)** and **SO1-3(c)**

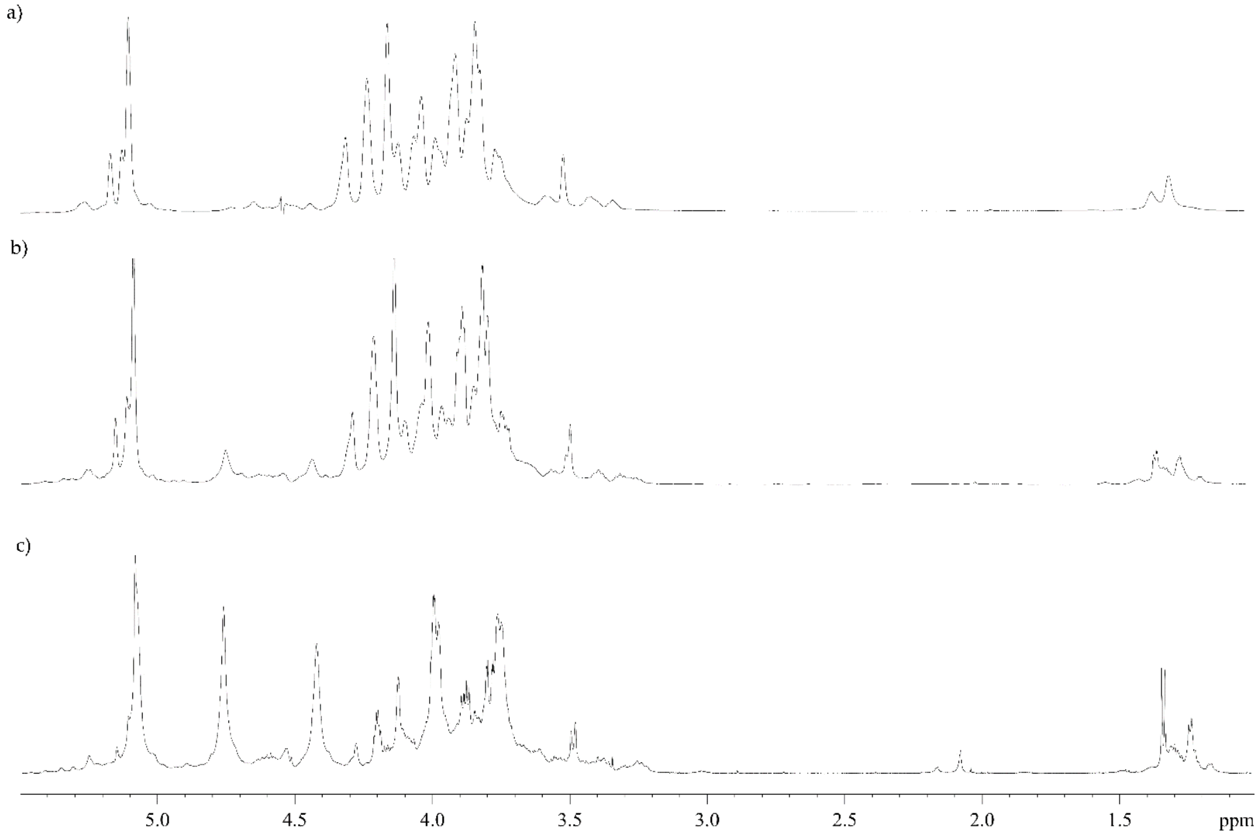

Figure S2. Part of ^{1}H, ^{13}C HMBC spectrum of the **SO1-3**.

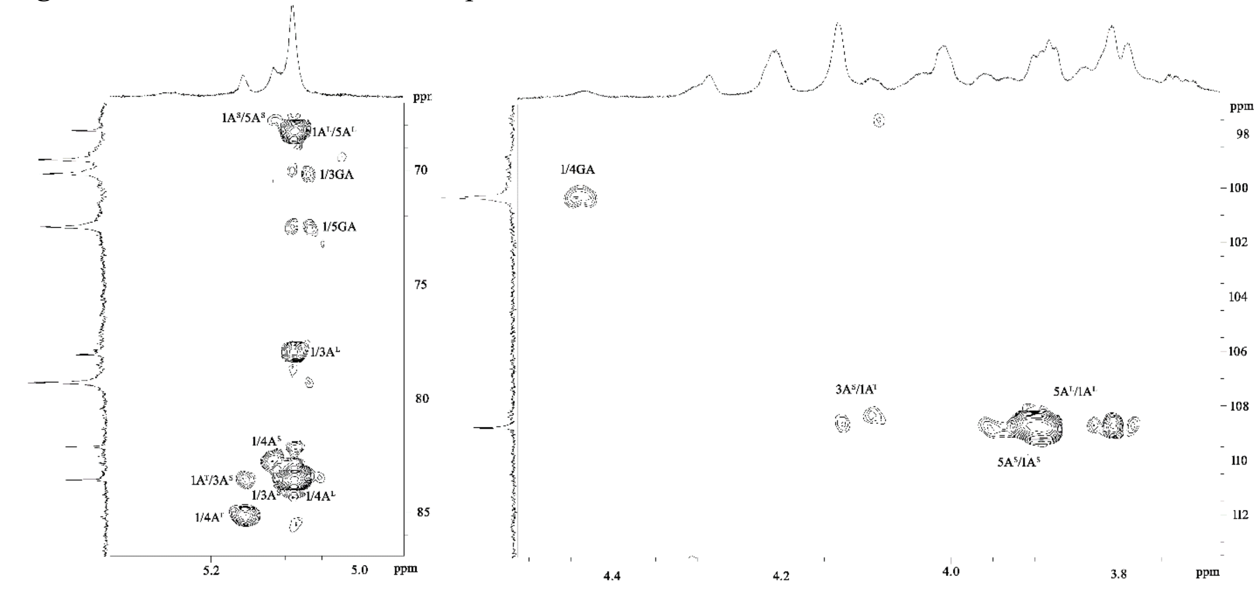

Figure S3. Part of ^{1}H, ^{1}H ROESY spectrum of the **SO1-1**.

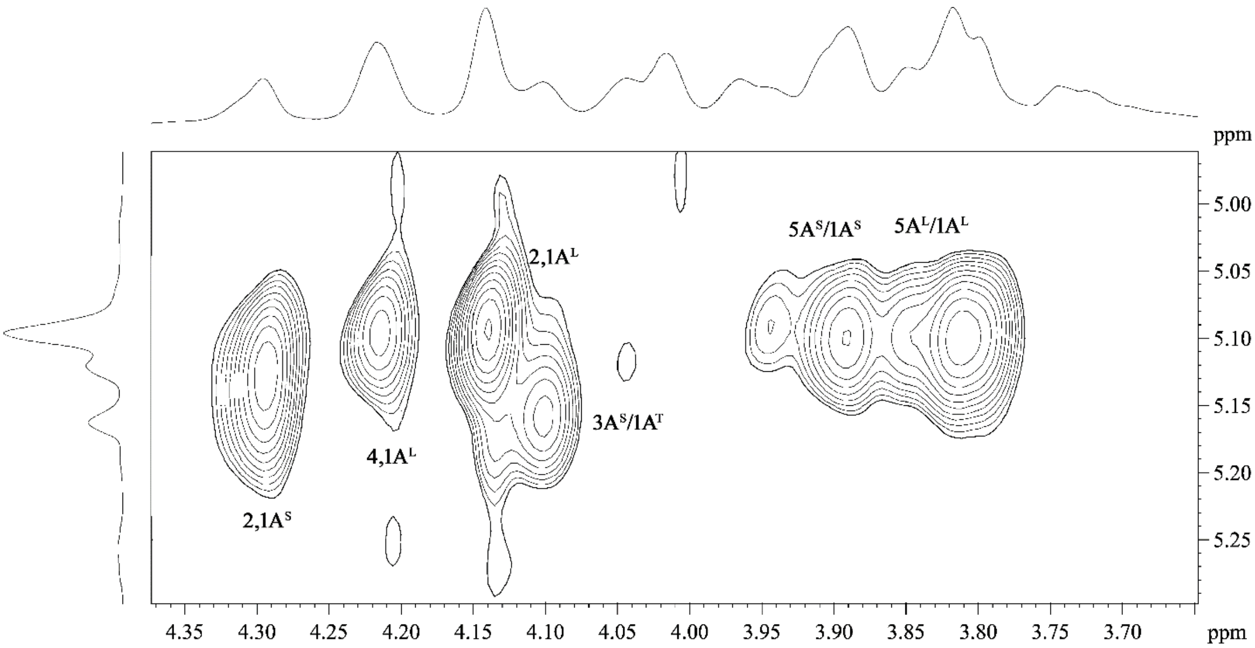

Figure S4. Part of ^{1}H, ^{1}H ROESY spectrum of the **SO1-2**.

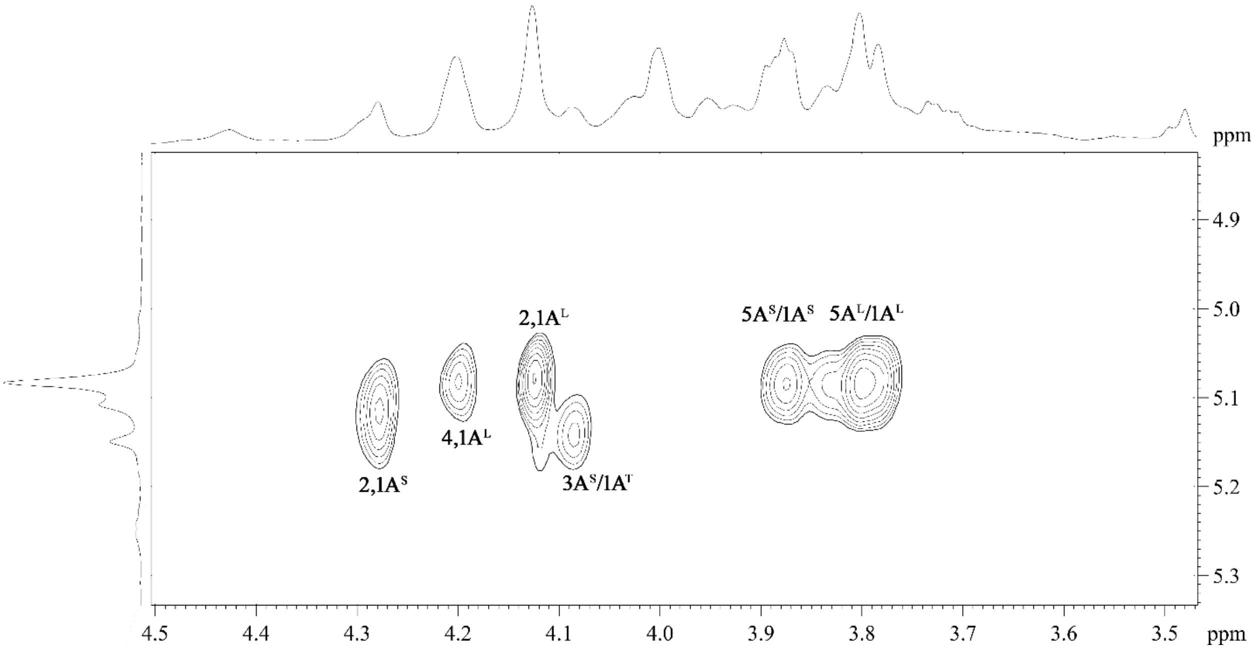

Supplement: Supplementary file 1 [file ijms-23-13175-s001.zip › ijms-1978783-supplementary.pdf]
